# A Closer Look at Obesogens: Lipid Homeostasis Disruption in *Daphnia*

**DOI:** 10.1289/ehp.123-A219

**Published:** 2015-08-01

**Authors:** Lindsey Konkel

**Affiliations:** Lindsey Konkel is a New Jersey–based journalist who reports on science, health, and the environment.

Obesogenic chemicals promote weight gain in mammals by altering lipid metabolism, which results in increased fat accumulation.^1^ Altered lipid metabolism has been associated with serious health problems, including obesity, diabetes, and an increased risk of cardiovascular disease.^2^ However, very little is known about how obesogenic chemicals might affect invertebrate species. In this issue of *EHP*, researchers suggest that altered lipid transport from the maternal organism to the egg by a known obesogen may be responsible for reproductive problems in *Daphnia magna*, a tiny freshwater crustacean.^3^

In the study, the researchers exposed *Daphnia* to 0.1 µg/L or 1.0 µg/L of the organometallic pollutant tributyltin (TBT). Once used widely as a biocide in paints designed to keep marine invertebrates from sticking to the hulls of ships, TBT was later shown to accumulate in the environment and harm the reproduction and development of aquatic organisms.^4^ In marine snails, TBT causes a condition called imposex, in which females develop male sex organs.^5^ In mouse studies it also has been shown to increase lipid accumulation and promote weight gain across multiple generations.^6^ In 2008 the International Maritime Organization severely restricted its use as a biocide.^4^

**Figure:**
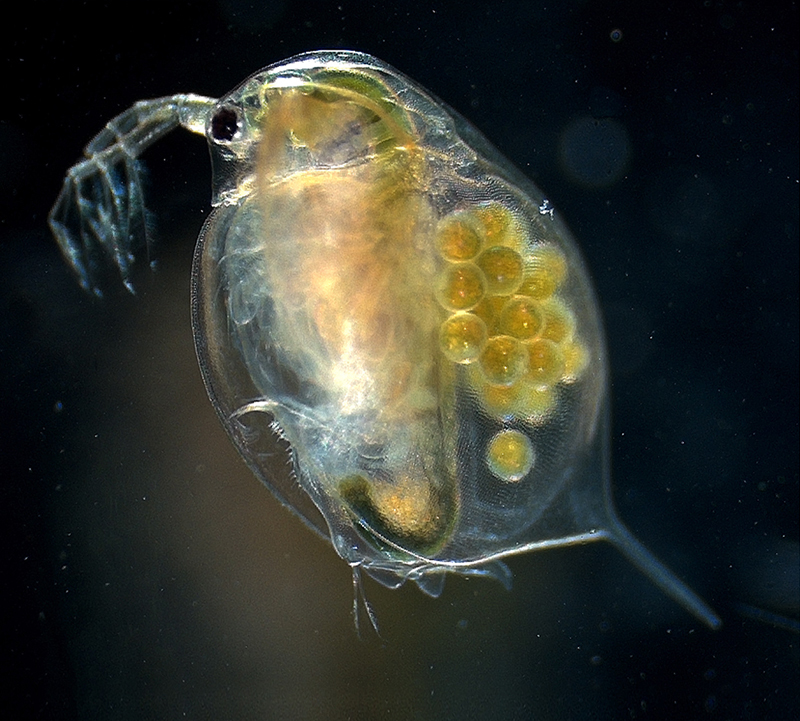
Experimental exposure to TBT altered the transport of lipids from mother Daphnia to her eggs, an effect that could be problematic if it were to occur in an ecological context © Hajime Watanabe/doi:10.1371/image.pgen.v07.i03

*Daphnia*, also known as the water flea, is used widely as a model in ecotoxicology studies.^7^ Within the last decade, scientists have started to use *Daphnia* in biomedical research as a surrogate species for genomic responses to environmental stressors.^7^ “We wanted to test whether *Daphnia* could be used as an invertebrate model for obesogenic effects,” says senior author Carlos Barata, an ecotoxicologist at the Institute of Environmental Assessment and Water Research in Barcelona, Spain.

Female water fleas typically release a brood of offspring each time they molt (shed their exoskeleton). Between molts, *Daphnia* stores up triacylglycerols from food as lipid droplets. Fats are allocated to the formation of a new carapace during the molting stage and are transferred into eggs; as this occurs, triacylglycerol levels drop.^8^

Barata and colleagues observed negative effects on reproduction and survival in *Daphnia* exposed to TBT. “Our findings suggest that TBT disrupted the normal transfer of lipids from adult to egg,” Barata says. Lipid levels in eggs from TBT-exposed females were lower than those in eggs from nonexposed females. Females exposed to TBT also retained more fat droplets after they molted. As adults, offspring of exposed females were less fit—they showed impaired survival and reduced reproduction.^3^

“We’ve known for a long time that TBT causes reproductive problems in *Daphnia*. This could be a mechanistic explanation,” says Gerald LeBlanc, an environmental toxicologist at North Carolina State University. LeBlanc was not involved in the study.

Previous research has suggested that TBT may disrupt certain hormonal signaling pathways in *Daphnia*.^9^ In this study, the researchers found evidence that TBT activated signaling pathways related to molting and reproduction, presumably by increasing transcription of the retinoid X receptor (*RXR*) gene.^3^ In vertebrates, the nuclear receptor known as peroxisome proliferator-activated receptor γ partners with the RXR to stimulate fat cell differentiation and lipid storage.^10^ These receptors are known targets for TBT.^11^

However, LeBlanc says it would be premature to call this a model organism for the study of obesogens. “The molecular pathways that are affected [by TBT] in vertebrates don’t exist in *Daphnia*,” he says.

Bruce Blumberg, a professor of developmental and cell biology at the University of California, Irvine, agrees it may be premature to call *Daphnia* a model for the study of obesogens. Obesogens are defined as chemicals that produce weight gain by increasing the number and/or size of adipocytes or by modulating lipid metabolism.^12^ Although lipid transfer is clearly important for fecundity of the animals, Blumberg points out that “the perturbation of lipid transfer from mother to egg in itself isn’t necessarily an obesogenic effect.” Blumberg was not involved in the current study.

Yet if chemicals that cause mammals to get fatter can also perturb lipid dynamics in an ecological context, there could be implications beyond obesity, says Michele La Merrill, a toxicologist at the University of California, Davis. “Although this study is not evaluating invertebrate obesity, it is showing some changes in lipids, and that might be a problem since lipids are important for a number of cell functions, such as membrane integrity,” she explains. La Merrill was not involved in the study.

Barata hopes the research will spur more scientists to explore the roles of known obesogens and emerging contaminants in invertebrates. He says, “There may be new mechanisms of toxicity that can affect our environment.”

## References

[1] Grün F Blumberg B Minireview: the case for obesogens. Mol Endocrinol 23 8 1127 1134 2009; 10.1210/me.2008-0485 19372238 PMC2718750

[2] Grün F Blumberg B Perturbed nuclear receptor signaling by environmental obesogens as emerging factors in the obesity crisis. Rev Endocr Metab Disord 8 2 161 171 2007; 10.1007/s11154-007-9049-x 17657605

[3] Jordão R Obesogens beyond vertebrates: lipid perturbation by tributyltin in the crustacean *Daphnia magna*. Environ Health Perspect 123 8 813 819 2015; 10.1289/ehp.1409163 25802986 PMC4529017

[4] IMO. International Convention on the Control of Harmful Anti-fouling Systems on Ships [website]. London, United Kingdom:International Maritime Organization (2015). Available: http://www.imo.org/en/About/Conventions/ListOfConventions/Pages/International-Convention-on-the-Control-of-Harmful-Anti-fouling-Systems-on-Ships-(AFS).aspx [accessed 14 July 2015]

[5] Iguchi T Developmental effects: oestrogen-induced vaginal changes and organotin-induced adipogenesis. Int J Androl 31 2 263 268 2008; 10.1111/j.1365-2605.2008.00863.x 18248399

[6] Chamorro-García R Transgenerational inheritance of increased fat depot size, stem cell reprogramming, and hepatic steatosis elicited by prenatal exposure to the obesogen tributyltin in mice. Environ Health Perspect 121 3 359 366 2013; 10.1289/ehp.1205701 23322813 PMC3621201

[7] NIH. Model Organisms for Biomedical Research: *Daphnia* [website]. Bethesda, MD:National Institutes of Health (2015). Available: http://www.nih.gov/science/models/daphnia/ [accessed 14 July 2015]

[8] Tessier AJ, Goulden CE (1982). Estimating food limitation in cladoceran populations.. Limnol Oceanogr.

[9] Wang YH Tributyltin synergizes with 20-hydroxyecdysone to produce endocrine toxicity. Toxicol Sci 123 1 71 79 2011; 10.1093/toxsci/kfr154 21673327

[10] Pagliarani A Toxicity of organotin compounds: shared and unshared biochemical targets and mechanisms in animal cells. Toxicol in Vitro 27 2 978 990 2013; 10.1016/j.tiv.2012.12.002 23232461

[11] Santos MM, et al. Lipid homeostasis perturbation by organotins: effects on vertebrates and invertebrates. In: Biochemical and Biological Effects of Organotins (Pagliarani A, Trombetti F, Ventrella V, eds.). Bologna, Italy:Bentham Science Publishers (2012)

[12] Grün F Blumberg B Environmental obesogens: organotins and endocrine disruption via nuclear receptor signaling. Endocrinology 147 6 suppl S50 S55 2006; 10.1210/en.2005-1129 16690801

